# Digital planning of orthognathic surgery with aligners: ortho-facial-driven, a viable protocol for surgery-first treatment

**DOI:** 10.1590/2177-6709.30.5.e25spe5

**Published:** 2026-01-09

**Authors:** Bruno Nehme BARBO, Fabiane AZEREDO, Susana Maria Deon RIZZATTO, Guilherme Gehner FRITSCHER, Fernando ANDRIOLA, Luciane Macedo de MENEZES

**Affiliations:** 1 Pontifical Catholic University of Rio Grande do Sul, School of Health and Life Sciences, Department of Orthodontics (Porto Alegre/RS, Brazil).; 2 Pontifical Catholic University of Rio Grande do Sul, Department of Oral and Maxillofacial Surgery (Porto Alegre/RS, Brazil).

**Keywords:** Orthognathic surgery, Removable orthodontic appliances, Orthodontics, Cirurgia ortognática, Alinhadores estéticos, Ortodontia

## Abstract

**Introduction:**

Traditionally, orthodontic-surgical treatment is performed in three stages: presurgical orthodontics, orthognathic surgery and post-surgical orthodontics. Though predictable and stable, this protocol requires a long treatment time and can temporarily compromise the patient’s aesthetics. Early benefit surgery eliminates or reduces the initial stage of orthodontic treatment, providing more immediate aesthetic and functional improvement.

**Objective:**

To present a virtual diagnosis and planning protocol, combining cone beam computed tomography (CBCT) and digital models applied to early benefit surgeries using orthodontic aligners.

**Method:**

Ten patients with dentofacial deformities underwent the proposed protocol. The accuracy of virtual planning was assessed by comparing 30 planned cephalometric measurements with those achieved postoperatively, on the “x” (transverse), “y” (anteroposterior) and “z” (vertical) axes, using anatomical reference points.

**Results:**

Most measurements did not present statistically significant differences between planning and the surgical outcome. The exceptions were the incisive foramen on the z axis (mean difference = -1.79, P=0.045) and the left gonion on the x axis (mean difference = -1.52, P=0.029).

**Conclusion:**

The proposed early benefit surgery protocol with aligners proved to be a viable, safe and effective approach to conventional orthodontic-surgical treatment, with good clinical predictability rates.

## RESUMO

**Introdução:**

Tradicionalmente, o tratamento ortodôntico-cirúrgico é realizado em 3 etapas: ortodontia pré-cirúrgica, cirurgia ortognática e ortodontia pós-cirúrgica. Apesar de previsível e estável este protocolo requer longo tempo de tratamento, e pode comprometer temporariamente a estética do paciente. A cirurgia de benefício antecipado elimina ou reduz a etapa inicial do tratamento ortodôntico proporcionando uma melhora estética e funcional mais imediata.

**Objetivo:**

Apresentar um protocolo de diagnóstico e planejamento virtual, combinando tomografia computadorizada de feixe cônico (TCFC) e modelos digitais aplicado a cirurgias de benefício antecipado com o uso de alinhadores ortodônticos.

**Método:**

dez pacientes com deformidade dentofaciais foram submetidos ao protocolo proposto. A acurácia do planejamento virtual foi avaliada pela comparação de 30 medidas cefalométricas planejadas e as obtidas no pós-operatório, nos eixos “x” (transversal), “y” (antero-posterior) e “z” (vertical) utilizando pontos anatômicos de referência.

**Resultados:**

A maioria das medidas não apresentou diferenças estatisticamente significativas entre o planejamento e o resultado cirúrgico. As exceções foram o forame incisivo no eixo z (diferença média de -1,79, P=0,045) e o gônio esquerdo no eixo x (diferença média de -1,52, P=0,029).

**Conclusão:**

O protocolo de cirurgia de benefício antecipado com alinhadores proposto demonstrou ser uma abordagem viável, segura e eficaz ao tratamento ortodôntico-cirúrgico convencional, com bons índices de previsibilidade clínica.

## INTRODUCTION

The close relationship between orthodontist and oral and maxillofacial surgeon provides a significant advance in the quality of corrections of dentofacial deformities.^1^ ^,^ ^2^ Traditionally, orthodontic-surgical treatment is performed in three stages, starting with presurgical orthodontic treatment, followed by orthognathic surgery and finalizing with post-surgical orthodontic treatment. Though stable and predictable, it demands a long period and causes temporary aesthetic damage to the patient during the first stage until the surgical stage. Early benefit surgery, known as “Surgery-First”, was introduced in 2009 by Nagasaka et al.^3^, eliminating the initial treatment stage and performing the surgical procedure before orthodontics, aiming to improve the patient’s quality of life.^4^

The introduction of Cone Beam Computed Tomography (CBCT) and digital models in dental practice has enabled virtual planning of orthognathic surgeries, combining tomography (which reproduces an accurate image of skeletal and facial structures) with the digital model (which presents excellent detailing of the dental region).^5^ ^-^ ^7^ Another advantage of digital models is the possibility of fabrication of orthodontic aligners, initially used only in simple cases; however, with the technological advances, they have been used in more complex treatments. Currently, different possibilities of orthodontic-surgical treatments are presented, from surgery-first to traditional surgical treatments using orthodontic aligners.^8^

This paper aims to present a diagnostic and planning protocol with digital models and CBCT for use in Surgery-first associated with orthodontic aligners, as a predictable and effective option when carefully performed.

## TECHNIQUE

This clinical protocol for the diagnosis and planning of orthodontic- surgical treatments with surgery-first associated with orthodontic aligners was developed in the disciplines of Orthodontics and Oral and Maxillofacial Surgery of the School of Health and Life Sciences of the Pontifical Catholic University of Rio Grande do Sul (PUCRS, Brazil). The proposal aims to standardize the interdisciplinary approach, promoting greater predictability, organization of clinical flow and efficiency of outcomes.

As part of the presurgical planning protocol, extraoral photographs (frontal view, 45º and right and left lateral views, at rest and smiling) and intraoral photographs (upper and lower occlusal views, frontal, right and left lateral occlusion) are achieved, besides intraoral scanning and full-face CBCT. The tomography is achieved without the chin support to avoid interference in soft tissue imaging. Examinations are performed with exposure parameters to allow adequate three-dimensional resolution with the lowest possible radiation dose. When necessary, a bilateral jig is used to help maintaining the centric relationship during CBCT acquisition. The lower and upper arches and occlusion record are scanned, generating digital models that are later transferred to image manipulation software in STL (stereolithography) format. The digital models are used both for preparing the setups (orthodontic treatment planning) and for planning orthognathic surgery. This protocol used the OrthoAnalyzer Software (3Shape, Copenhagen, Denmark) and Dolphin software (Dolphin Imaging & Management Solutions, Chatsworth, CA, USA) for digital planning.

Several multidisciplinary teams adopt different protocols, adapted to their clinical reality, to enable effective orthodontic-surgical planning. In the context of anticipated benefit surgery, three main approaches can be used: 1) orthodontically guided (orthodontic-driven),^9^ in which skeletal problems are corrected by surgery, seeking the best bone position, regardless of dental occlusion, which is corrected later by orthodontics; 2) surgically guided (surgery-driven),^9^ in which surgical and orthodontic problems are solved by orthognathic surgery, based on the best possible dental stability; and 3) guided by orthodontics-surgery collaboration (Ortho-Facial-Driven), which is recommended in this protocol and jointly considers facial aesthetics, limits of tooth and bone movements and functional stability to define the treatment plan.

Some occlusal aspects are essential to determine the feasibility of surgery-first. Manipulation of study models (digital, printed or plaster) in simulated post-surgical occlusion allows the identification of possible occlusal interferences, such as undesirable contacts or transverse discrepancies that compromise the occlusal stability. In these situations, it is necessary to perform orthodontic movements before surgery, as described by Uribe et al.^10^ and Liou et al.^11^, precluding surgery at this stage. The accomplishment of surgery-first in a predictable and stable manner requires previous resolution of such interferences. Once a stable occlusion has been established, without interferences that could compromise the surgical procedure, a new intraoral scan is performed. At this stage, both the current occlusion and the desired post-surgical occlusal relationship are recorded (this simulation can be performed by manipulating the models or directly in the software, if possible). Based on this new interarch relationship, the orthodontic setup is performed, in which the orthodontist defines the desired position of teeth at treatment completion.

**Figure 1:**
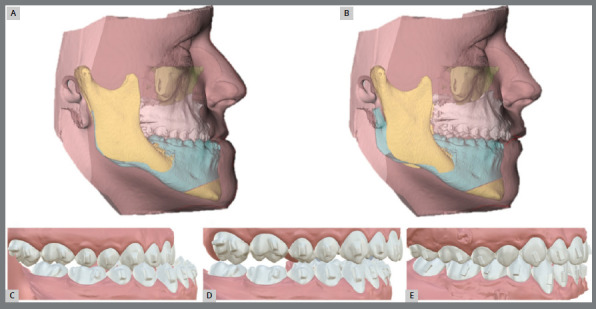
Virtual orthodontic-surgical planning. **A)** Pre-surgical Facial soft tissue. **B)** Surgery planning. **C)** Pre-surgical occlusion. D) Surgical planning occlusion. **E)** Orthodontic setup.

After accomplishment of the orthodontic setup, CBCT acquisition, facial analysis, and simulation of post-surgical occlusion and position, the team evaluates the final position of facial soft tissues. The main recommended reference is the final position of upper and lower incisors in relation to the facial soft tissue, since the combination of soft tissue thickness and dentoskeletal factors directly influences the harmony of the lower facial third^12^ (Fig. 1).

Planning includes cephalometric analysis before and after the surgical setup, with emphasis on the measurements U1.NA; U1-NA; L1.NB; L1-NB; U1.L1; IMPA; Mx1.MxOP; Md1.MdOP; and exposure of the upper incisors^8^. It should be considered that the final position of incisors will be corrected after surgery, according to the movements defined in the orthodontic setup.

At completion of orthodontic-surgical planning, the objective is to achieve predictable facial results, based on the following references:

»**Nasolabial angle:** defined by the nasal columella and the most anterior point of the upper lip, which should range from 85º to 105º. This value is influenced by both the position and inclination of the upper incisor and the nasal columella.^13^
»**Lip projection in relation to the Sn’-Pog’ line:** the upper lip should be on average 3.5 mm ± 1.4 mm in front of the Sn’-Pog’ line, while the lower lip should be 2.2 mm ± 1.6 mm in front of it.^14^ In the context of surgery-first, it is important to consider that orthodontic movement of upper and lower incisors will be performed after surgery. Palatal inclination of the upper incisor tends to increase the nasolabial angle and may reduce lip support. Studies indicate that, for every 1 mm of retraction of upper incisors, there is an average setback of 0.59 mm of the upper lip.^15^
»**Mentolabial angle:** formed by intersection of the lower lip and the chin, it has an average value of 120 ± 10º. In cases of excessively projected lower incisors and in Class II patients, there is eversion of the lower lip and reduction of the angle. In individuals with Class III malocclusion or retroclined lower incisors, the angle tends to be obtuse.^13^
»**Post-surgical occlusion:** the resulting occlusion should be adequate to allow achievement of the objectives proposed in the orthodontic setup, ensuring predictability and stability of results after the surgical procedure.

Planning should be discussed and modified until the objectives proposed by the surgeon and orthodontist are in harmony. Once defined in the treatment plan, the digital file of models is imported into the surgical planning software and fused to teeth in the tomography, following the workflow. The superimposition of virtual models on the tomographic reconstructions (sagittal, axial and coronal planes) must be carefully checked for accuracy.

Based on facial analysis, skeletal discrepancies and predicted dental movements, virtual manipulation of the bone bases is performed. The objective is to correct dentofacial deformities, maintain or improve the airway and achieve facial harmony, using the models as reference in the post-surgical occlusion. The simulation of surgical movements is initiated by the maxilla, following the concepts described by Reyneke and Ferretti^13^, in which positioning of the upper central incisor is the key point for adequate positioning of the maxillary bone. Based on the correct maxillary positioning, the mandible is adjusted to achieve the post- surgical occlusion previously defined by the team (using the software’s Piggyback tool). At this stage, the result is assessed, i.e., whether correction of the dentofacial deformity has brought about the expected benefits in skeletal, aesthetic and functional aspects (including airway volume), besides checking for possible bone interferences caused by the movement of segments.

After planning has been discussed again and accepted by the team and patient, the surgical guides are generated in STL format and 3D printed, serving as essential instruments for accurate transfer of surgical planning to the operating room.

After planning is finalized and approved, the patient is ready for surgery.

After maxillary osteotomy, intermaxillary guides are used to establish the intermediate and final occlusal position, enabling internal fixation with plates and screws. The absence of brackets and orthodontic arches during the procedure allows greater retention of the surgical guide on teeth, providing greater postoperative dental stability and minimizing undesired dental movements. Intraoperative intermaxillary block is performed with steel wire anchored in fixation screws preferably placed between the roots of second premolar and first molar, and between the lateral incisor and canine, bilaterally, in both arches. On the first postoperative day, the patient remains without block to avoid discomfort and the risk of blood aspiration. From the second day onwards, light elastics are used to guide the new occlusion and can be kept for up to two weeks. In the third week, the use of elastics is restricted to nighttime and, in the fourth week, the patient uses only the final guide, to adapt to the new occlusion and for correct mandibular positioning, considering that the post-surgical occlusion tends to present instability. From the fifth week onwards, the patient is released to begin orthodontic treatment.

Patients with Class III malocclusion usually present retroclination of lower incisors and proclination of upper incisors, while Class II patients tend to present proclination of lower incisors and retroclination of upper incisors. Thus, in the adopted protocol, when necessary, Class III patients complete surgery with a Class II canine relationship, while Class II patients complete the surgical stage with anterior edge-to-edge relationship, allowing dental decompensation and preventing relapses. When the incisors are already well-positioned in relation to the bone bases, surgery is performed using canine guidance.

**Figure 2:**
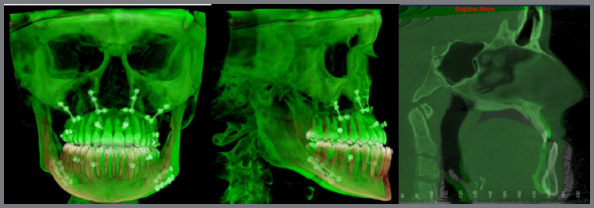
Accuracy of achieved results, assessed after superimposition. Pre-surgical tomography, color beige. Post-surgical tomography, color green, it is possible to visualize the 4 fixation screws in each quadrant used for intermaxillary fixation.

This methodology was applied in ten cases, after approval by Institutional Review Board of PUCRS (CAAE: 12407519000005336). No complications were observed during planning or during surgeries. All patients underwent postoperative CBCT, and the superimposition of pre- and post-surgical CT scans was performed using Dolphin software^16^ ^,^ ^17^ (Fig. 2).

To assess the accuracy of virtual surgical planning and the results achieved, the planned cephalometric measurements were compared with those achieved postoperatively. For that purpose, the accuracy of linear measurements on the “x” (transverse), “y” (anteroposterior), and “z” (vertical) axes was assessed using selected reference points that represented the various movements and effects resulting from surgery. Of the 30 pre- and post- surgical measurements compared, no statistically significant differences were observed in the 10 patients, except for two: the measurement at the incisive foramen on the z axis (mean difference -1.79, P=0.045), and of the left gonion on the x axis (mean difference -1.52, P=0.029). All means were below 2 mm, indicating acceptable clinical accuracy for application of the orthodontic-surgical planning protocol with aligners proposed in this study.

**Figure 3:**
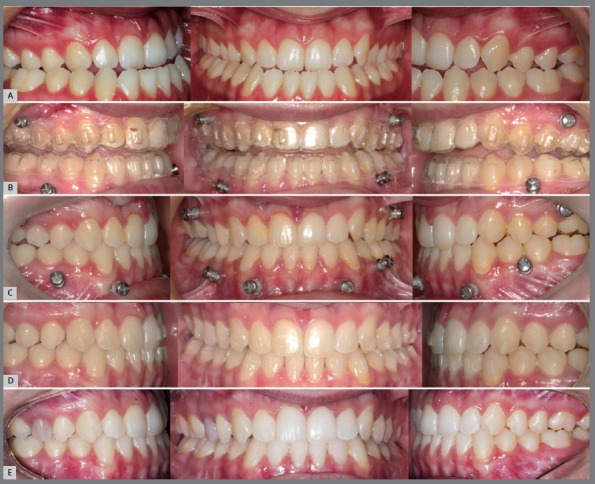
Stages of the occlusal relationship during treatment. **A)** Pre-surgical. **B)** Post-surgical. **C)** After the first phase of aligner treatment. **D)** Treatment completion. **E)** One year follow-up after treatment completion.

**Figure 4:**
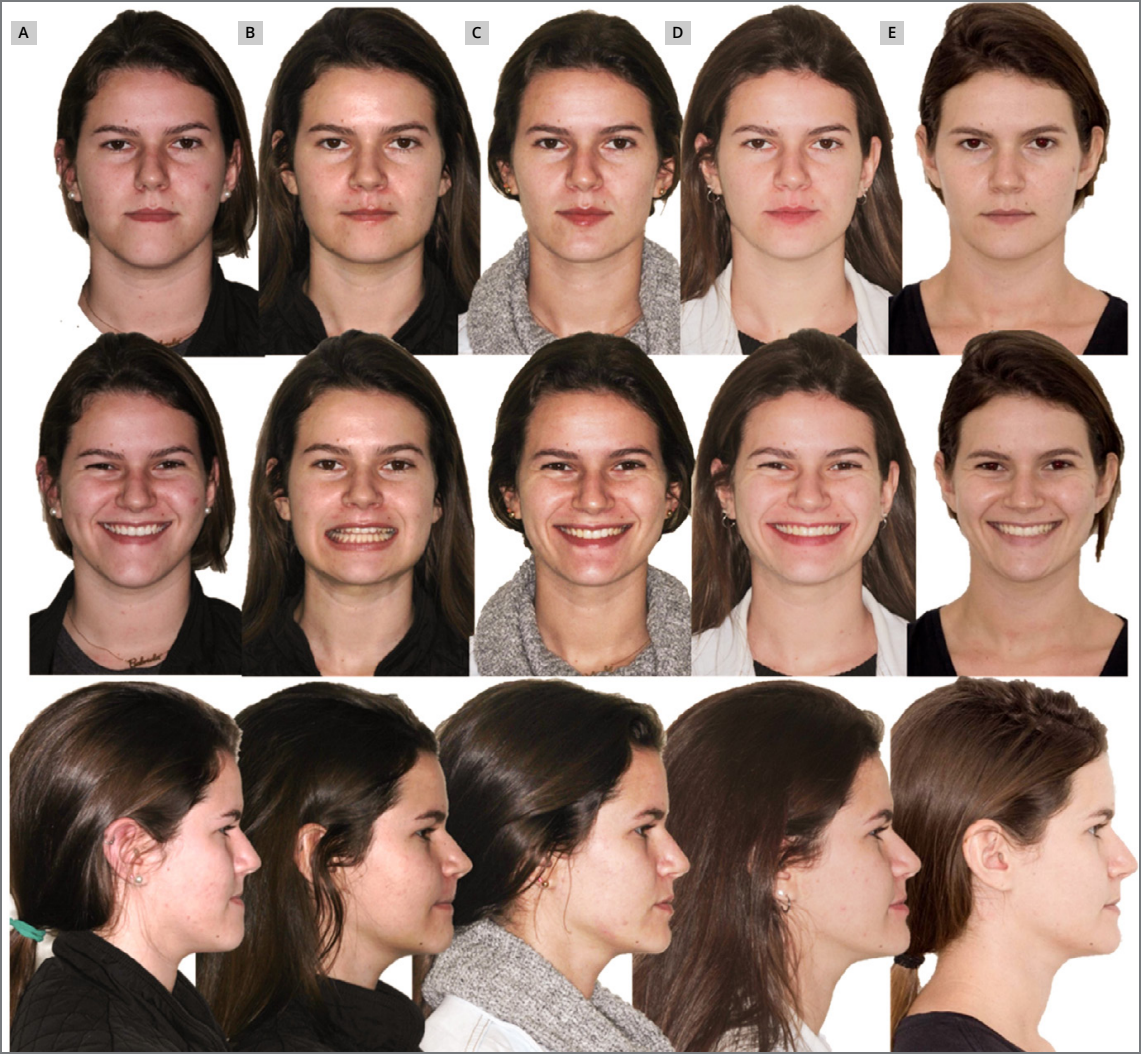
Facial photographs during treatment. **A)** Pre-surgical. **B)** Post-surgical. **C)** After the first phase of aligner treatment. **D)** Treatment completion. **E)** One year follow-up after treatment completion.

When the patient is released to begin orthodontic treatment, the anteroposterior relationships, when in Class II or in edge-to-edge relationship, are corrected with the aid of skeletal anchorage devices (miniplates or on the fixation screws themselves), installed during the surgical procedure. Once the Class I canine relationship has been established, a new virtual planning is performed to determine the necessary movements for orthodontic finalization. Figures 3 and 4 illustrate the evolution of treatment of a case with Class III malocclusion, from the presurgical stage to follow-up one year after treatment completion.

The protocol of change of aligners follows the specific recommendations of the system used, with intervals ranging from 7 to 15 days. In the final phase, intermaxillary elastics supported by buttons bonded to the teeth are used, or the aligners are used at night to improve the interarch occlusal relationship.

## DISCUSSION

All stages of the protocol for surgery-first with aligners developed at PUCRS are performed using CAD/CAM technology, except for the preparation of jigs to obtain the centric relation, since this stage is performed prior to planning.

The authors recommend continuing to scan the manually defined occlusion, as proposed by Nadjmi et al.^18^ and Lee et al.^19^, regardless of whether the models are plaster or printed. Although the color maps of the virtual occlusion may not show clinically relevant differences for diagnosis in some cases, the importance of caution is emphasized in situations that require greater accuracy and occlusal stability - essential factors for the success of orthodontic-surgical planning, especially when orthodontic aligners are used.

One of the critical aspects of virtual planning is the superimposition of digital models on CBCT. In the present protocol, the absence of orthodontic brackets and arches eliminates image artifacts, favoring a more accurate and adequate superimposition of dental arches and increasing the reliability of planning. The comparative analysis between virtual planning and the surgical results achieved demonstrated that all means of linear discrepancies remained within the 2-mm limit, considered clinically acceptable for orthognathic procedures.^20^ These findings confirm the accuracy of the proposed protocol.

The surgery-first has advantages over traditional orthognathic surgery, such as elimination of the prior orthodontic preparation phase. This reduces the initial aesthetic impact, accelerates the achievement of aesthetic-functional outcomes and improves the quality of life (QoL).^4^ ^,^ ^21^ Additionally, tooth movement tends to be faster in the postoperative period, as already demonstrated in the literature.^3^ ^,^ ^22^

Another essential aspect for consolidation of new therapeutic approaches is the occlusal stability achieved at treatment completion. Kwon et al.^23^ conducted a randomized clinical trial on patients with different dentofacial deformities, both in anteroposterior, vertical and transverse directions, who underwent orthodontic-surgical treatment with aligners. The authors observed a significant improvement in the PAR Index (Peer Assessment Rating) scores, both in relation to alignment and occlusal aspects, demonstrating the effectiveness of this orthodontic-surgical treatment method.

Orthodontic aligners have gained popularity due to their greater comfort, better aesthetics, easy cleaning, and the possibility of removal by the patient when necessary. Considering these advantages, as well as the clinical results observed in the 10 cases herein presented, it is concluded that the application of orthodontic aligners in the context of surgery-first brings several advantages for patients. When well indicated and carefully planned, they provide adequate and stable orthodontic-surgical results, similar to those achieved by the traditional method.^24^

## CONCLUSION

The surgery-first protocol with aligners proposed in this study proved to be a feasible, safe, and clinically effective approach. The results achieved demonstrate the potential of this technique as a predictable and consistent option to conventional orthodontic-surgical treatment.
